# Murine Pancreatic Acinar Cell Carcinoma Growth Kinetics Are Independent of Dietary Vitamin D Deficiency or Supplementation

**DOI:** 10.3389/fonc.2017.00133

**Published:** 2017-06-28

**Authors:** James Dooley, Vasiliki Lagou, Nathalie Heirman, Tom Dresselaers, Uwe Himmelreich, Adrian Liston

**Affiliations:** ^1^Translational Immunology Laboratory, VIB, Leuven, Belgium; ^2^Department of Microbiology and Immunology, KU Leuven, Leuven, Belgium; ^3^Department of Imaging and Pathology, KU Leuven, Leuven, Belgium

**Keywords:** pancreatic cancer, vitamin D, *in vivo*, pancreatic acinar carcinoma, magnetic resonance imaging

## Abstract

Vitamin D has been proposed as a therapeutic strategy in pancreatic cancer, yet evidence for an effect of dietary vitamin D on pancreatic cancer is ambiguous, with conflicting data from human epidemiological and intervention studies. Here, we tested the role of dietary vitamin D in the *in vivo* context of the well-characterized Ela1-TAg transgenic mouse model of pancreatic acinar cell carcinoma. Through longitudinal magnetic resonance imaging of mice under conditions of either dietary vitamin D deficiency (<5 IU/kg vitamin D) or excess (76,500 IU/kg vitamin D), compared to control diet (1,500 IU/kg vitamin D), we measured the effect of variation of dietary vitamin D on tumor kinetics. No measurable impact of dietary vitamin D was found on pancreatic acinar cell carcinoma development, growth or mortality, casting further doubt on the already equivocal data supporting potential therapeutic use in humans. The lack of any detectable effect of vitamin D, within the physiological range of dietary deficiency or supplementation, in this model further erodes confidence in vitamin D as an effective antitumor therapeutic in pancreatic acinar cell carcinoma.

## Introduction

Vitamin D has been proposed as a potential therapeutic for pancreatic cancer, a disease for which efficacious treatments are currently lacking; however, the evidence for an impact of vitamin D on pancreatic cancer is ambiguous. The most consistent evidence for vitamin D as an antitumor drug comes from cell line work. Vitamin D has been shown to be effective at inhibiting the proliferation of pancreatic cancer cell lines *in vitro*, including the active form 1α,25-dihydroxyvitamin D3 (1, 2), the prohormone 25-hydroxyvitamin D(3) (3), and the analogs calcipotriol (4, 5), 22-oxa-1,25-dihydroxyvitamin D3 (5), EB 1089 (2, 6, 7), MART-10 (8), 1,25-dihydroxyvitamin D(3)-3-bromoacetate (9), 19-nor-1 alpha,25-dihydroxyvitamin D2 (paricalcitol) (10), and 22- oxa-1,25-dihydroxyvitamin D3 (maxacalcitol) (11). The antiproliferative effect has even been observed *in vivo*, when pancreatic cancer cell lines have been transplanted into immunodeficiency mice (2, 5, 7, 8, 10, 11). It should, however, be noted that this effect is not universal, as when multiple cell lines have been tested, it is only a minority that respond to vitamin D *in vitro* (5, 10, 11). In addition, the complex relationship between tumor, stroma, and the immune system in lost under these testing conditions, so the results in cell lines may not reflect the physiological impact on patients.

Epidemiological studies on the interaction between vitamin D and pancreatic cancer have proven inconsistent. A clear correlation of pancreatic cancer risk has been associated with increasing latitude and lower ultraviolet B (UVB) radiation, known to induce vitamin D production. A protective effect of UVB dose is observed across countries (12, 13) and within individual countries (14–17), and a similar correlation shows reduced risk in individuals with fair skin color (15). A simple explanation of this phenomenon would be that vitamin D production protects against pancreatic cancer, yet the evidence for this is underwhelming. The best surrogate for vitamin D levels is circulating 25-hydroxyvitamin D. Cohort analysis for pancreatic cancer risk have variously identified either low (18, 19) or high (20, 21) plasma 25-hydroxyvitamin D as a risk factor for developing pancreatic cancer, with a meta-analysis indicating no significant association (22). Following pancreatic cancer development, analysis of a prospective cohort found reduced survival in patients who had insufficient levels of plasma 25-hydroxyvitamin D (23), yet a retrospective analysis of other cohorts found no link with survival (24, 25). Likewise studies have found either no link (26) or only a weak protective link (27) between plasma levels of vitamin D-binding protein, the primary carrier of 25-hydroxyvitamin D, and pancreatic cancer risk. A link between vitamin D receptor (VDR) polymorphisms and pancreatic cancer risk has been observed in the Chinese population (28), but other large studies have found no link with any of the genes in the vitamin D pathway (23, 29). Together, these studies place doubt over the link between endogenous vitamin D and pancreatic cancer risk, without excluding a potential benefit for exogenous administration.

In contrast to the large number of epidemiological studies investigating the link between pancreatic cancer and vitamin D, relatively few interventional trials have been performed. A phase II trial of EB 1089 found no antitumor effect (30), while a phase II trial of docetaxel gave a modest effect in three patients (31). Larger studies have been performed with dietary vitamin D supplementations, with two studies indicating an increased risk of pancreatic cancer after supplementation (32, 33), but a meta-analysis of nine studies finding overall no significant association of dietary vitamin D with pancreatic cancer (22). Overall, despite the positive results from pancreatic cancer cell lines, the limited epidemiological and intervention studies performed leave the status of vitamin D as a potential therapeutic for pancreatic cancer in doubt. Here, we have sought to formally test the role of dietary vitamin D in the *in vivo* context through utilization of a well-characterized animal model of pancreatic acinar cell carcinoma, the Ela1-TAg transgenic mouse. Through longitudinal magnetic resonance imaging (MRI) assessment of Ela1-TAg transgenic mice under conditions of either vitamin D deficiency or dietary excess, we measured the effect of vitamin D on spontaneous tumor development, growth rates, and mortality. The lack of any detectable effect of vitamin D deficiency or dietary supplementation in this model further erodes confidence in vitamin D as an effective antitumor therapeutic in pancreatic acinar cell carcinoma.

## Materials and Methods

### Mice

Ela1-TAg mice, expressing the SV40 large T Antigen under the control of the Elastase-1 acinar cell promoter, were purchased from Jackson on the C57BL/6 background (34). Mice were bred under specific pathogen-free conditions and from the time of breeder setup were exclusively fed on either ssniff^®^ EF R/M Control chow (1,500 IU/kg vitamin D, 0.9% Calcium; “control diet”), ssniff^®^ EF R/M Vitamin D3-deficient chow (<5 IU/kg vitamin D; “Vitamin D deficient diet,” 0.9% Calcium), or ssniff^®^ EF R/M Vitamin D3 excess chow (76,500 IU/kg vitamin D, 1.59% Calcium; “Vitamin D excess diet”). Mice were moved to conventional conditions at 7 weeks of age (maintaining special diets) for longitudinal MRI. All experimental protocols were approved by the University of Leuven Animal Ethics Committee, and all experiments were performed in accordance with the guidelines and regulations from the University of Leuven Animal Ethics Committee. Mouse weight and blood glucose were monitored throughout.

### Imaging

Mice were scanned under isoflurane anesthesia using a Bruker Biospin 9.4 T Biospec small animal MR scanner (Bruker Biospin, Ettlingen, Germany). The scanner was equipped with an actively shielded gradient set of 600 mT/m using a respiration triggered spin echo sequence (RARE) with 50 continuous slices of 0.5 mm thickness in interlaced mode (acquisition parameters: repetition time = 6,000 ms, echo time = 15.9 ms, field of view = 4.0 cm × 6.0 cm, a matrix of 200 × 400, two dummy scans, and two averages). For radio-frequency irradiation and detection, a 7.2-cm quadrature resonator was used.

### Data and Statistical Analysis

Magnetic resonance imaging scans were analyzed with ImageJ (National Institute of Health, Bethesda, MD, USA) and the mean area at maximum radius was used to infer volume using the formula: 4/3*area*√(area/π). Statistical analysis was performed in R (https://www.r-project.org/ version 3.1.2). Cumulative incidence curves were generated using the R package “survplot” with the fun = function (*x*) {1 − *x*} argument (35). Survival curves were generated using the Kaplan–Meier method implemented in the R “survplot” package, and statistical analysis was performed using log-rank test implemented in the R “survdiff” package (36).

## Results

**Figure 1:**
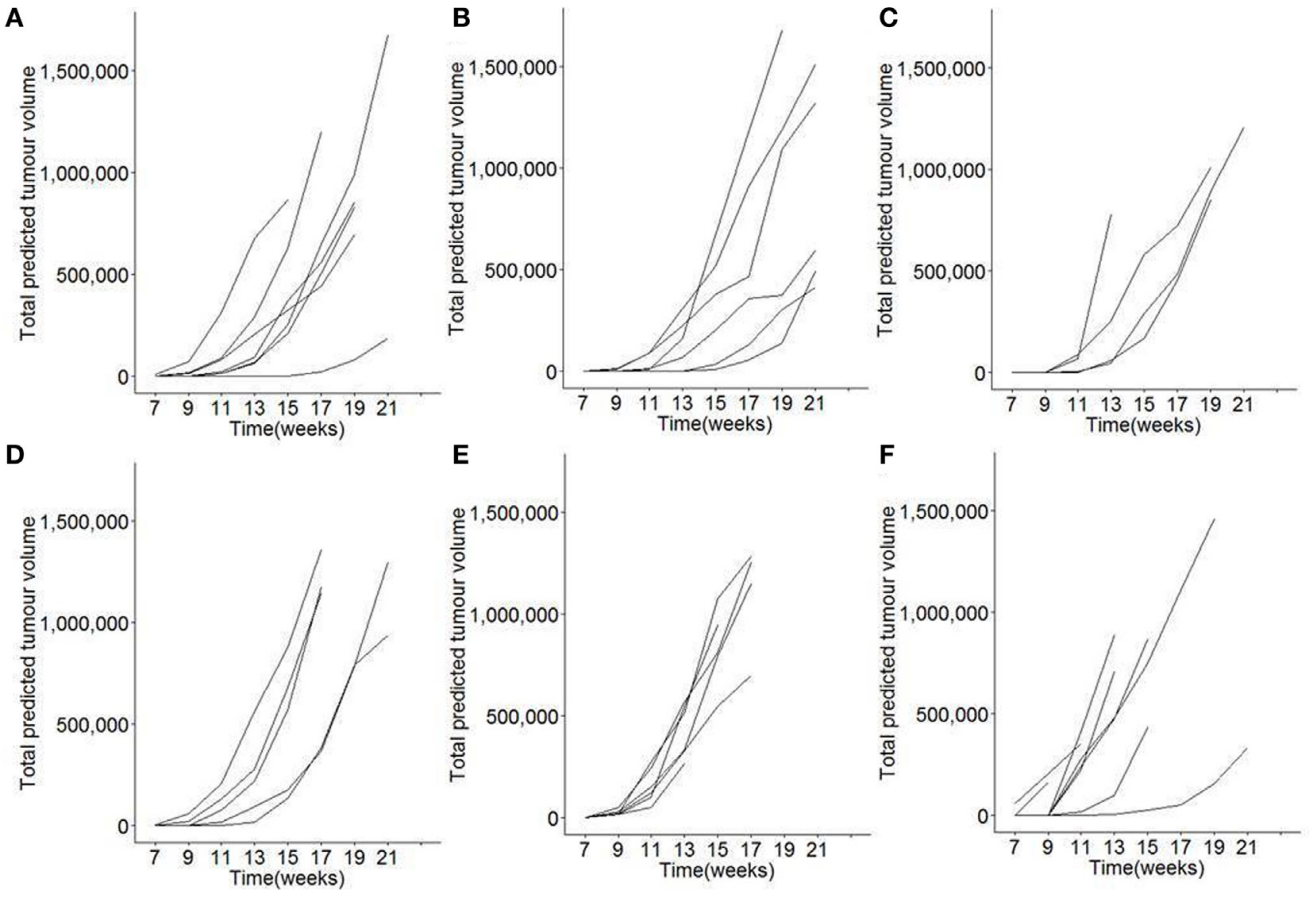
Longitudinal monitoring of tumor growth following modification of dietary vitamin D in mice. TAg^+^ mice were placed on either control, vitamin D deficient, or vitamin D excess diets *in utero* and aged on the same diets to 21 weeks of age. From 7 weeks onward, mice were assessed through Magnetic Resonance Imaging for tumor size. Individual total predicted tumor volume curves for **(A)** female mice on control diet (*n* = 7), **(B)** female mice on vitamin D-deficient diet (*n* = 6), **(C)** female mice on vitamin D excess diet (*n* = 4), **(D)** male mice on control diet (*n* = 5), **(E)** male mice on vitamin D-deficient diet (*n* = 6), and **(F)** male mice on vitamin D excess diet (*n* = 9).

In order to systematically test the role of dietary vitamin D in pancreatic acinar cell carcinoma development, growth, and mortality, we utilized the well-characterized Ela1-TAg transgenic mouse model, where expression of the SV40 large T Antigen results in the spontaneous formation of pancreatic tumors from acinar cells (34). To maximize the differences in vitamin D exposure, breeder cages were setup on either control diet (1,500 IU/kg vitamin D3), vitamin D-deficient diet (<5 IU/kg vitamin D3), or vitamin D excess diet (76,500 IU/kg vitamin D3). This study design allows alteration of dietary vitamin D levels from the *in utero* condition onward, with mice being weaned onto the corresponding diet. From 7 weeks of age, each mouse underwent MRI scanning for tumor detection and volume estimation, revealing exponential growth of tumors after first detection (Figure 1).

**Figure 2:**
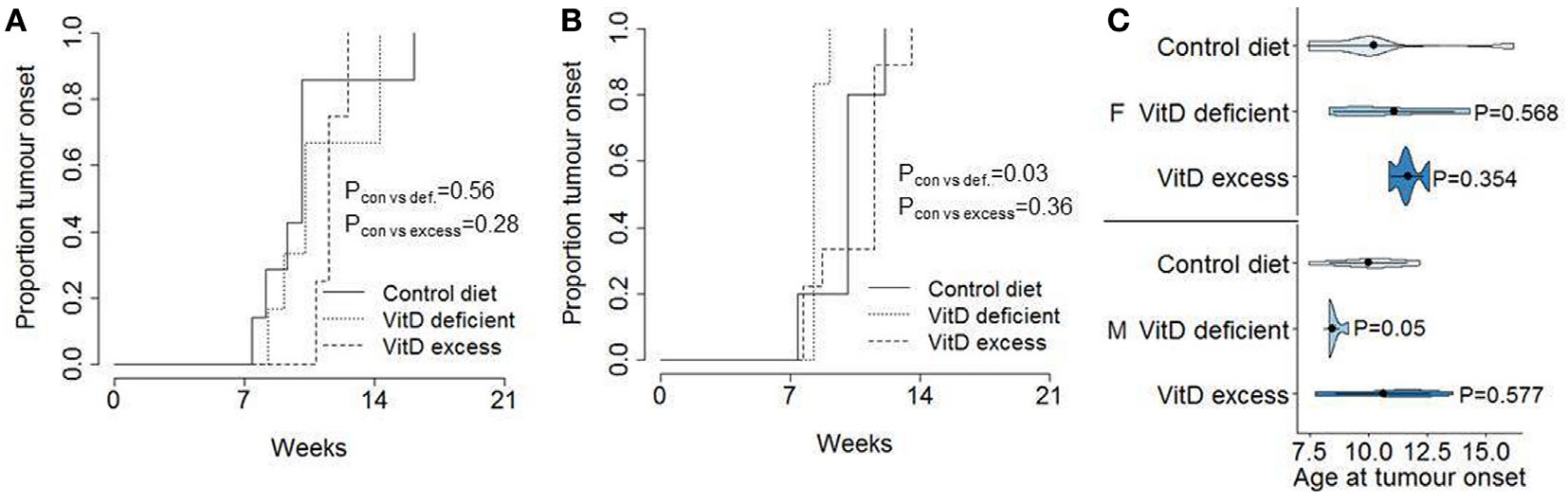
No consistent effect of dietary vitamin D on tumor onset in a pancreatic cancer model. TAg^+^ mice were placed on either control, vitamin D-deficient, or vitamin D excess diets *in utero* and aged on the same diets to 21 weeks of age. Cumulative incidence of pancreatic cancer was recorded as a function of age at tumor detection, in **(A)** female (*n* = 7, 6, 4) and **(B)** male (*n* = 5, 6, 9) mice. The *P* values were calculated using the log-rank test. **(C)** Violin plots showing the mean, SD, and kernel probability density of the age at tumor onset under each condition. The *P* values were calculated using two-tailed unpaired *t* test.

To determine the effect of dietary vitamin D on initial tumor development, the age of first tumor detection was assessed. Reliable tumor detection was achieved for tumors >3 mm in size (data not shown). For female mice, no significant difference was observed in the cumulative incidence of pancreatic acinar cell carcinoma (Figure 2A). For male mice, marginal significance was observed for mice fed a vitamin D-deficient diet having earlier tumor onset (Figure 2B); however, this result was not significant when age of first tumor detection was directly tested (Figure 2C). It is also notable that the data from female mice did not even follow the same trend, indicating that there is no consistent effect of dietary vitamin D, either in deficiency or in supplementation, on tumor onset.

**Figure 3:**
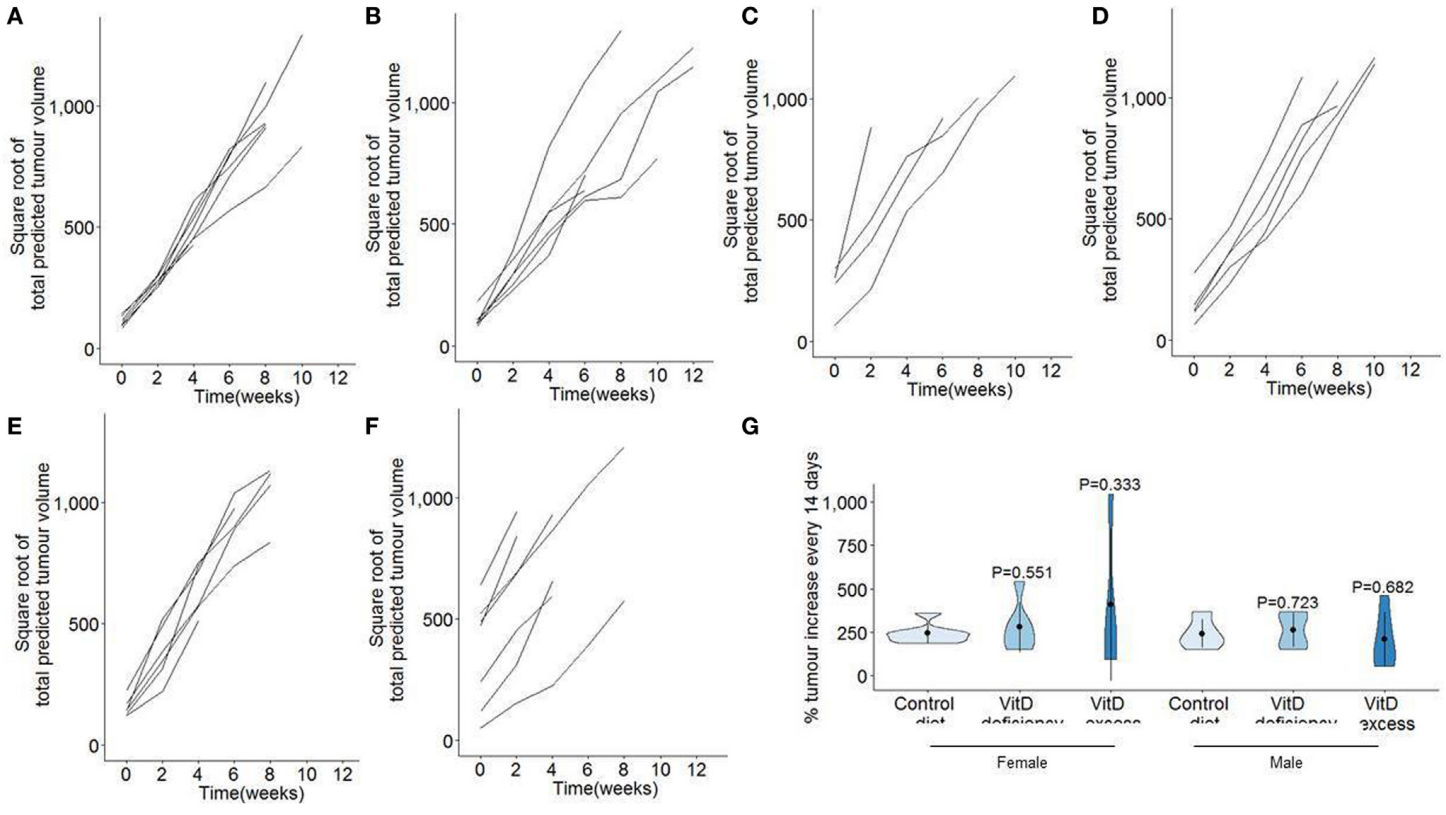
No effect of dietary vitamin D on tumor growth in a pancreatic cancer model. TAg^+^ mice were placed on either control, vitamin D-deficient or vitamin D excess diets *in utero* and aged on the same diets to 21 weeks of age. From 7 weeks onward, mice were assessed through Magnetic Resonance Imaging for tumor size. Total predicted tumor volumes were square root transformed and normalized to age of first detection for **(A)** female mice on control diet (*n* = 7), **(B)** female mice on vitamin D-deficient diet (*n* = 6), **(C)** female mice on vitamin D excess diet (*n* = 4), **(D)** male mice on control diet (*n* = 5), **(E)** male mice on vitamin D-deficient diet (*n* = 6), and **(F)** male mice on vitamin D excess diet (*n* = 9). **(G)** Violin plots showing the mean, SD, and kernel probability density of the percentage of tumor volume increase every 2 weeks. The *P* values were calculated using two-tailed unpaired *t* test.

We next assessed the growth rate of the established tumor burden. Due to the variable onset and exponential growth rates (Figure 1), total tumor volume estimates from MRI were square root transformed and plotted from time of first detection (Figures 3A–F). Linear plots on the transformed graphs indicate that once established the tumors exhibited a consistent growth rate until experimental end-point. This analysis allows tumor growth to be calculated as the percentage increase in total tumor volume every 14 days (i.e., time between MRI assessment), averaged over the entire period of monitoring. Using this criteria, no difference was observed in tumor growth rates in either males or females, under conditions of either vitamin D dietary deficiency or supplementation (Figure 3G).

**Figure 4:**
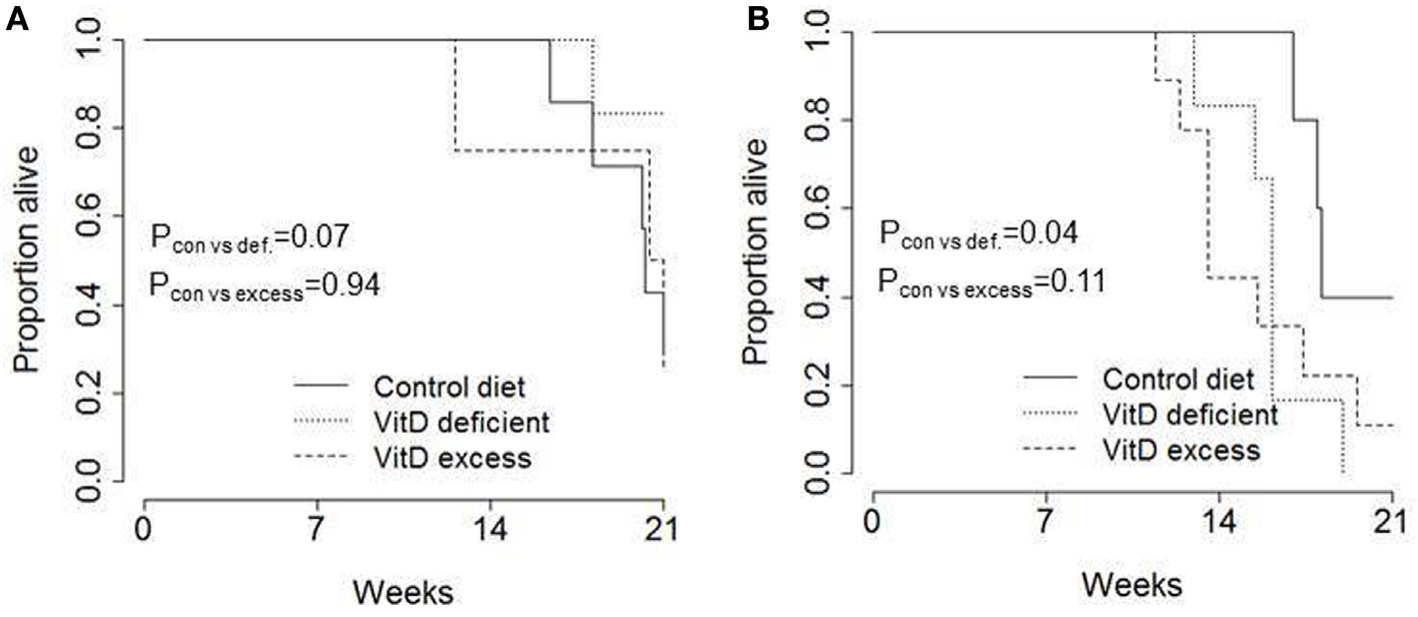
No consistent impact of dietary vitamin D on survival in a transgenic pancreatic cancer model. TAg^+^ mice were placed on either control, vitamin D-deficient or vitamin D excess diets *in utero* and aged on the same diets to 21 weeks of age. Kaplan–Meier plots showing the overall pancreatic cancer survival in **(A)** female (*n* = 7, 6, 4) and **(B)** male (*n* = 5, 6, 9) mice on control, vitamin D-deficient and vitamin D excess diets. The *P* values were calculated using the log-rank test.

Finally, we assessed the impact that changes to dietary vitamin D had on tumor-induced mortality (Figure 4). On the control diet, 50% of mice had reached end-point (condition deteriorating to end-point guidelines) by 20 weeks in female mice (Figure 4A) and 18 weeks in male mice (Figure 4B). No significant alteration was observed in female mice by changing the vitamin D content of the diet (Figure 4A). In male mice, an increase in mortality was observed in mice fed a vitamin D-deficient diet (Figure 4B); however, the significance was marginal compared to control diet and there was no difference between vitamin D deficiency and vitamin D excess. Overall, these results do not indicate a convincing impact of vitamin D on mortality in this murine model of pancreatic acinar cell carcinoma.

## Discussion

Direct comparisons between the dietary doses used here and the recommended dietary intakes in humans are complicated by species differences. The European Food Safety Authority recommends 200 IU/day intake for adults, with an upper intake of 4,000 IU/day. The diets used here result in consumption of 0.02 IU/day (Vitamin D deficient), 5 IU/day (control), and 250 IU/day (Vitamin D excess). These values, however, do not take into account the body weight difference between mice and humans; normalized to average human body weight, the daily intakes range from far below recommended intake (30 IU/day/70 kg for Vitamin D-deficient diet) to far above the upper intake limit (9,000 IU/day/70 kg for control diet and 460,000 IU/day/70 kg). The most relevant comparison, however, is probably the resulting circulating serum 1α,25-dihydroxyvitamin D3 level. In adult humans, the desirable level of 1α,25-dihydroxyvitamin D3 is 20 ng/ml, while ostemolacia or rickets develops at levels lower than 10 ng/ml, adverse effects are reported at levels above 80 ng/ml and toxicity at 150 ng/ml and greater (37–39). In mice, diets with vitamin D levels below 125 IU/kg result in serum levels of 1α,25-dihydroxyvitamin D3 in the sub-20 ng/ml range, diets 1,000–5,000 IU/kg result in serum 1α,25-dihydroxyvitamin D3 in the 20–40 ng/ml range, 125,000 IU/kg causes 150–200 ng/ml serum 1α,25-dihydroxyvitamin D3, 250,000–500,000 IU/kg causes >600 ng/ml serum 1α,25-dihydroxyvitamin D3 and hypercalcemia, while >1,000,000 IU/kg results in toxicity (40–42). An approximate cross-species comparison would, therefore, have the “Vitamin D deficient” diet paralleling Vitamin D deficiency in humans, the “control” diet resulting in serum 1α,25-dihydroxyvitamin D3 in the recommended range for humans, and the “Vitamin D excess” diet resulting in the serum 1α,25-dihydroxyvitamin D3 above the recommended range for humans and approaching the threshold for adverse effects.

The lack of any measurable impact of vitamin D on pancreatic acinar cell carcinoma development, growth, or mortality in the TAg mouse model casts further doubt on the already equivocal data supporting potential therapeutic use in humans. There are, however, several pertinent caveats to be made, including the use of a single mouse model [as the low-throughput nature of mouse models prohibits parallel screening (43)], potential differences between acinar cell carcinomas and other pancreatic tumors, and the species barrier. Nevertheless, while there are distinct limitations to mouse models for pancreatic cancer, they also have distinct advantages over analysis of cell lines. The generation of pancreatic cancer cell lines necessitates a selection for independence from micro-environmental cues and stromal interactions. In effect, any molecule which inhibits proliferation will exhibit antitumor impacts in these systems, regardless of additional more complex pro-tumor effects which may negate the antitumor properties. Vitamin D and its analogs are thought to directly inhibit proliferation by increasing p21 and p27 (11, 44) and inhibiting FOXM1 (2) and the Wnt/β-catenin signaling pathway (4). *In vivo*, other, opposing, cues may come into consideration, such as the effect of vitamin D in countering retinoic acid-induced apoptosis (45) and the tolerogenic impact vitamin D has on the immune system (46). This latter effect may have profound pro-tumor consequences, and yet the interaction will be absent not only in *in vitro* models but also in the xenograft transplantations, due to the use of immunocompromised mice. The most parsimonious reconciliation of the cell line and mouse model work is that the antiproliferative impact of vitamin D is negated *in vivo* by these additional pro-tumor effects.

How then to account for the inconsistent associations found between serum vitamin D and pancreatic cancer? One plausible explanation lies in the known risks factors of pancreatic cancer, which include smoking, lack of physical activity, obesity, type 2 diabetes, and chronic pancreatitis (47). In several studies, smoking has been demonstrated to reduce serum 1α,25-dihydroxyvitamin D3 levels (48–50). Likewise, increased body mass index (BMI), an (imperfect) measure of obesity, is consistently associated with reduced serum 1α,25-dihydroxyvitamin D3 (51). Lower 1α,25-dihydroxyvitamin D3 is also found in patients with either type 2 diabetes (52) or pancreatitis from various sources (53). Increased 1α,25-dihydroxyvitamin D3 is also associated with increased exercise, although the effect is difficult to entangle from increased exposure to UVB and reduced BMI (54). In any case, lower vitamin D levels are directly associated with many of the major risk factors for pancreatic cancer. Thus, it is possible that the inconsistent association of vitamin D with pancreatic cancer is actually a reflection of these indirect (and non-causative) associations.

The lack of support for an anti-oncogenic role of vitamin D in pancreatic cancer stands in contrast to that observed in several other solid tumors. In colorectal cancer, for example, vitamin D (55, 56) and various vitamin D analogs (57, 58) have proven efficacious as antitumorigenic agents in multiple mouse models. Supporting this function, genetic ablation of VDR in mice increases development of colorectal cancers (59, 60). Beyond pancreatic cancer the evidence from humans is also stronger. Epidemiological data from human studies support a protective role for vitamin D in colorectal cancer and bladder cancer, with elevated circulating 1α,25-dihydroxyvitamin D3 levels reducing cancer risk (61). For other cancer types, the epidemiological data remains equivocal, or, in the case of prostate cancer, even identifies a negative association (61), although there are suggestive protective associations with several. The effect of vitamin D on cancer may also be broader than risk of development; in breast cancer, for example, circulating 1α,25-dihydroxyvitamin D3 is only weakly associated with risk, but is strongly associated with survival (62). At a genetic level, polymorphisms in VDR are associated with total risk of developing cancer (63), with the strongest effects being on skin cancer and gynecological cancers (63, 64), while several other cancers show no consistent association (although stratification by ethnicity and sex can identify specific risk groups). Our negative results in pancreatic cancer should not, therefore, be taken as evidence against the protective nature of vitamin D in other cancers, although it does caution against excessively broad characterization of vitamin D as a generic panacea for cancer.

Finally, we would note that even direct extrapolation of our study to the human context, which would not be advisable without the study of additional models, would not completely preclude therapeutic potential of vitamin D. First, our study was performed on acinar cell carcinoma, and it is plausible that other, more common, forms of pancreatic cancer may respond differently to vitamin D. Second, we used dietary modification with classical vitamin D, while therapeutic approaches in patients may include the use of alternative delivery systems and modified vitamin D analogs capable of more potent biological effects. Third, VDR was recently identified as a gemcitabine sensitizer in pancreatic cancer cells in an *in vitro* screen (65), and in a study of transplanted murine tumor cells into immuno-competent mice the vitamin D analog calcipotriol enhanced the antitumor effect of gemcitabine (66). Thus even in the absence of a monotherapeutic *in vivo* antitumor effect, there is potential for vitamin D to exhibit utility as a component of a combination therapy.

## Ethics Statement

All experimental protocols were approved by the University of Leuven Animal Ethics Committee, and all experiments were performed in accordance with the guidelines and regulations from the University of Leuven Animal Ethics Committee.

## Author Contributions

JD and AL designed the study. JD, NH, TD, and UH performed experiments. VL analyzed the data. AL wrote the manuscript, which was reviewed and approved by all authors.

## Conflict of Interest Statement

The authors declare that the research was conducted in the absence of any commercial or financial relationships that could be construed as a potential conflict of interest.
